# Drug discovery and treatment paradigms in nonalcoholic steatohepatitis

**DOI:** 10.1002/edm2.105

**Published:** 2019-12-10

**Authors:** Mazen Noureddin, Mark D. Muthiah, Arun J. Sanyal

**Affiliations:** ^1^ Division of Digestive and Liver Diseases Comprehensive Transplant Center Cedars Sinai Medical Center Los Angeles California; ^2^ Department of Medicine Yong Loo Lin School of Medicine National University of Singapore Singapore; ^3^ Division of Gastroenterology and Hepatology National University Hospital National University Health System Singapore; ^4^ Division of Gastroenterology, Hepatology and Nutrition Virginia Commonwealth University School of Medicine Richmond Virginia

**Keywords:** drug development, drug therapy, nonalcoholic steatohepatitis

## Abstract

Nonalcoholic fatty liver disease (NAFLD) is the most common chronic liver disease in western populations, and is closely associated with features of the metabolic syndrome. The burden of disease is set to rise exponentially, and this is further compounded by the lack of good medications. In addition, these patients tend to have multiple comorbidities that may not be adequately managed. In this article, we review the biological basis of potential therapies in nonalcoholic steatohepatitis (NASH), the current drugs being tested in clinical trials, as well some practical considerations in managing patients in the clinic.

## Graphical abstract

In this article, we review the biological basis of potential therapies in NAFLD, the current drugs being tested in clinical trials, as well some practical considerations in managing patients in the clinic.

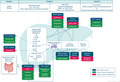

## 1 INTRODUCTION

Nonalcoholic fatty liver disease (NAFLD) affects almost a third of the Western population, and the burden of the disease is set to rise exponentially.^1^ Current drug therapies for the resulting nonalcoholic steatohepatitis (NASH) are limited in their efficacy, and there are no FDA‐approved drugs for NASH.^2^ In this article, we review the potential therapeutic targets for NASH and their biological rationale, the current state of drugs in clinical trials, as well as some practical considerations as to how we manage NASH in the clinic.

## 2 TREATMENT TARGETS FOR THERAPEUTICS IN NASH

### 2.1 Targeting homeostasis of lipid and carbohydrate metabolism

**Figure 1:**
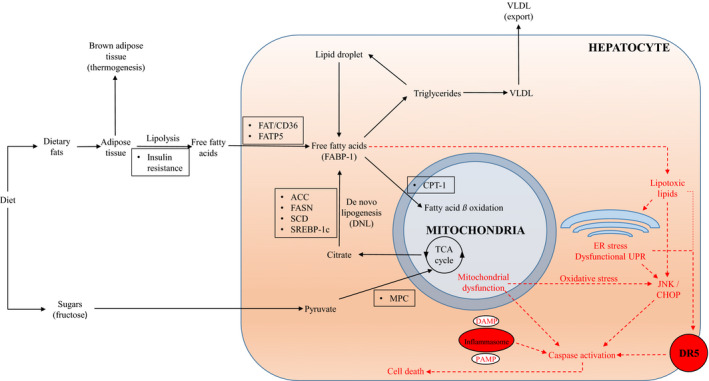
Mechanisms of intrahepatic glucose and lipid homeostasis leading to lipotoxicity and cell death. Intrahepatic free fatty acids are key to the development of NASH. Dietary fat is stored in adipocytes as triglycerides. They undergo lipolysis, and the free fatty acids are taken up into the liver. The two major fates of free fatty acids are to undergo beta oxidation in the mitochondria, or re‐esterification to triglycerides, where they are stored as lipid droplets or repackaged and excreted as VLDL. The triglycerides on lipid droplets can undergo lipolysis to form free fatty acids. When these two disposal pathways get overwhelmed, lipotoxic lipids form. This leads to oxidative stress, ER stress and recruitment of pro‐apoptotic receptors. With prolonged ER stress, a dysfunctional UPR occurs. Either DAMPs from breakdown products, or PAMPs from bacterial products can activate the inflammasome. All of these can lead to activation of caspases, which in turn lead to cell death. In boxes are key mediators of some of the processes, and in red are the pathogenic processes leading to cell death. ACC, acetyl Co‐A carboxylase; CHOP, CAATT enhancer binding homologous protein; CPT‐1, carnitine palmitoyltransferase 1; DAMP, danger‐associated molecular patterns; DNL, de novo lipogenesis; DR5, death receptor 5; ER, endoplasmic reticulum; FABP‐1, fatty acid binding protein‐1; FASN, fatty acid synthetase; FAT/CD36, fatty acid translocase/CD36; FATP5, fatty acid transport protein 5; JNK, c‐Jun N‐terminal kinase; MPC, mitochondrial pyruvate carrier; NASH, nonalcoholic steatohepatitis; PAMP, pathogen‐associated molecular proteins; SCD, stearoyl CoA‐desaturase; SREBP‐1c, sterol regulatory element binding protein‐1c; TCA, tricarboxylic acid; UPR, unfolded protein response; VLDL, very low‐density lipoprotein

Excess delivery of fatty acids to the liver or impaired disposal of fatty acids from the liver leads to excess energy supply. Hepatocytes are unable to process this excess energy, forming lipotoxic metabolites with hepatocellular injury.^3^ Systemic insulin resistance contributes to this, via increased adipose lipolysis leading to increased delivery of free fatty acids to the liver.^4^ Fatty acid transport protein 5 (FATP5) and fatty acid translocase CD36 (FAT/CD36) are involved in the uptake of these fatty acids into the liver.^5^, ^6^ Fatty acids in the liver can also arise from de novo lipogenesis (DNL), especially from fructose‐based carbohydrate substrates.^7^, ^8^ These free fatty acids in the liver are bound to fatty‐acid binding protein‐1 (FABP‐1) and either undergo beta oxidation in the mitochondria, or re‐esterification to form triglycerides. The formed triglycerides are released in the blood as very low‐density lipoprotein (VLDL) or stored in lipid droplets. The triglycerides on lipid droplets can undergo subsequent lipolysis, releasing free fatty acids back into the hepatocyte. However, when these disposal methods get overwhelmed by excess free fatty acids, they form lipotoxic lipids^9^ (Figure 1).

Consequently, a potential strategy in treating NASH is aiming to reduce the intrahepatic free fatty acids. This can be achieved by improving insulin sensitivity, reducing DNL, increasing beta oxidation of fatty acids or increasing export and storage in peripheral tissues. Peroxisome proliferator–activated receptor gamma (PPAR‐γ) agonism can help improve insulin sensitivity, can enhance adipocyte fat storage and has been shown to improve histology in NASH.^10^ Dipeptidyl peptidase‐4 (DPP‐4) inhibition and glucagon‐like peptide‐1 (GLP‐1) agonists also improve insulin sensitivity and may potentially be useful in NASH.^11^, ^12^

De novo lipogenesis can be targeted by inhibiting key enzymes such as acetyl‐CoA carboxylase (ACC), fatty acid synthetase (FASN) or stearoyl CoA‐desaturase (SCD).^13^, ^14^, ^15^ Sterol regulatory element binding protein‐1c (SREBP‐1c) is a major transcriptional regulator of other enzymes involved in DNL, including ACC, FASN, SCD, acetyl‐CoA synthetase (ACS), glycerol‐3‐phosphate acyltransferase (GPAT), ATP‐citrate lyase, malate dehydrogenase, glucose‐6‐phosphate dehydrogenase (G6PD) and 6‐phospgogluconate dehydrogenase (6PGD).^16^ SREBP‐1c can be potentially be targeted to antagonize DNL directly or indirectly. The farnesoid X receptor (FXR) and the liver X receptor (LXR) repress and activate SREBP‐1c, respectively, and provide the link between bile acid metabolism and DNL.^17^ FXR agonism can be targeted to help improve insulin sensitivity, inhibit DNL and inhibit bile acid synthesis. Obeticholic acid and fibroblast growth factor analogues have both demonstrated benefit in NASH patients via FXR agonism.^18^, ^19^ Antagonism of the mitochondrial pyruvate carrier (MPC) can prevent pyruvate from entering the mitochondrial matrix, preventing formation of acetyl‐CoA, which is required for carbohydrate substrates to undergo DNL.^20^ Other possible targets to inhibit DNL include FAT/CD36, FABP‐1 and 5′ adenosine monophosphate‐activated protein kinase (AMPK).^21^

Peroxisome proliferator–activated receptor alpha agonism increases the beta oxidation of fatty acids and is used in combination with PPAR‐δ or PPAR‐γ, which reduces inflammation and improves insulin sensitivity.^22^ Agonism of thyroid hormone receptors (THR) not only increases the beta oxidation of fatty acids, but potentially can modulate DNL as well as the uptake of fatty acids in the liver.^23^ Diverting the lipid load to peripheral tissues can help to reduce the intrahepatic free fatty acids. The TGR5 bile acid membrane receptor on brown adipose tissue can upregulate thermogenesis and may help with energy disposal.^24^ Other potential targets include carnitine palmitoyltransferase 1 (CPT‐1), which is involved in fatty acid beta oxidation.^25^

### 2.2 Targeting lipotoxicity and cell death

It is not clear which are the specific lipotoxic lipids in NASH, but candidates include saturated fatty acids, lysophosphatidyl choline, ceramide and free cholesterol.^26^ Lipotoxicity and hepatocellular injury lead to endoplasmic reticulum (ER) stress. This triggers the unfolded protein response (UPR), which initially helps to maintain homeostasis. With prolonged ER stress, the UPR becomes dysfunctional, leading to activation of apoptotic pathways.^27^ NASH is associated with mitochondrial dysfunction, causing alterations in the balance between pro‐ and anti‐oxidant mechanisms, producing reactive oxygen species (ROS) and oxidative stress.^28^, ^29^ Oxidative stress and ER stress can lead to the activation of the c‐Jun N‐terminal kinase (JNK) pathway, as well as the induction of CAATT enhancer binding homologous protein (CHOP).^30^ These lead to mitochondrial dysfunction, caspase release and apoptosis.^31^ Lipotoxicity and ER stress can also lead to increased death receptor 5 (DR5) expression, leading to recruitment of caspase 8 and apoptosis via activation of caspase 3, 6 and 7^32^ (Figure 1).

Pyroptosis, similar to apoptosis, acts in conjunction with inflammasome activation in the liver to play an important role linking lipotoxicity and cell death.^33^ The inflammasome is cytoplasmic complex that responds to danger‐associated molecular patterns (DAMPs) and pathogen‐associated molecular proteins (PAMPs). It leads to pyroptosis through activation of caspase 1.^34^

Targeting oxidative stress is a potential therapeutic target in NASH. In fact, Vitamin E, one of the few drugs available to treat NASH, targets oxidative stress.^35^ Given that cell death is the major consequence of lipotoxicity, targeting apoptosis or pyroptosis is a possible strategy in treating NASH.^36^ Targeting caspases, the effector pathways of both apoptosis and pyroptosis, may also be a potential drug target for NASH.^37^

Augmenting UPR‐mediated proteins activated in ER stress such as inositol requiring enzyme 1α/X‐box binding protein 1 (IRE1α/XBP1), protein kinase R‐like endoplasmic reticulum kinase/eukaryotic translation initiation factor 2 α (PERK/eIF2α) and activating transcription factor‐6 (ATF‐6) may help to ameliorate the damage from the ER stress.

Altering the proportion of lipotoxic lipids in the liver may be a potential strategy. With polyunsaturated fatty acids (PUFA), the ratio of omega‐6 to omega‐3 may play a role in NASH.^38^ Omega‐3 supplementation has been considered in the treatment of NASH, but benefit has been limited.^39^

### 2.3 Targeting inflammation

The innate immune system serves as a sensor to these stressors, recognizing endogenous cell death products (DAMPs) or bacterial products (PAMPs) via toll‐like receptors (TLRs) 2, 4 and 9.^40^, ^41^, ^42^ These not only contribute to the activation of the inflammasome, but also lead to activation of nuclear factor NF‐κB, JNK pathways, and signal the activation of transcription factors involved in the production of pro‐inflammatory cytokines, chemokines and interferons.^40^

Kupffer cells (KCs) play a major role in the recruitment of inflammatory cells in the injured liver, expressing C‐C motif chemokine receptors 2 and 5 (CCR2 and CCR5) on their surface when activated.^43^ The interaction of CCR2 with its ligand, CCL2, serves to enhance bone marrow–derived macrophage chemotaxis.^44^ These pro‐inflammatory macrophages are the first inflammatory cells present and a key cellular driver of the inflammation in NASH.^45^ They act in concert with tissue‐resident macrophages (Kuppfer cells) to drive the inflammatory process.^46^ In later stages of the disease, other inflammatory cell types are seen in the inflammatory milieu.^45^

Inflammation in the liver may also be triggered from extra‐hepatic sources. Adipokines from adipose tissue, such as leptin and adiponectin, may have pro and anti‐inflammatory effects on the liver.^47^ Patients with NASH have altered gut permeability and dysbiosis, which may give rise to inflammation in the liver via TLR4 and IL‐8.^48^, ^49^

In targeting inflammation to treat NASH, therapies may either target the pro‐inflammatory pathways or enhance pathways that terminate and resolve inflammation. In the former, pro‐inflammatory pathways involving ASK1, JNK, NF‐κB, ERK and MAP kinases are potent mediators of inflammation and potential therapeutic targets.^9^ Inhibiting recruitment of inflammatory cells (macrophages) into the liver is also a possible strategy.^50^ Inflammation can also be targeted by enhancing pro‐resolution pathways such as restorative macrophages or specialized pro‐resolving mediators (SPMs).^51^, ^52^ External sources of inflammation such as modulating the gut microbiome are an attractive strategy, but further studies are first needed to elucidate inter‐organ crosstalk.

### 2.4 Targeting fibrosis

Contrary to long‐held beliefs that fibrosis is not reversible, current thinking is that the liver is maintained in a balance between fibrogenesis and fibrolysis.^53^ This appears to be possible in NASH, with up to 20% of patients on the placebo arm of recent trials demonstrating regression of fibrosis.^54^ Fibrosis in the liver occurs with activation of the hepatic stellate cells (HSCs) to myofibroblasts after liver injury, with accumulation of extracellular matrix (ECM).^55^ These myofibroblast may also originate secondary to portal inflammation.^56^, ^57^ HSCs can be activated by fibrogenic cytokines, most notably transforming growth factor‐β (TGF‐β).^58^ HSCs can also be activated by HSC‐ECM interactions or Hedgehog pathway signalling.^59^, ^60^ The mechanisms and pathways implicated in fibrosis have been comprehensively reviewed elsewhere.^61^

The strategy to target fibrosis in NASH would be to either inhibit fibrogenesis or enhance fibrolysis. Regulating HSCs or their activation to myofibroblasts may be a possible strategy. Silencing heat shock protein 47 (HSP47) can enhance apoptosis of HSCs via ER stress.^62^ Inhibition of galectin‐3 abrogates fibrosis by inhibition of myofibroblast activation.^63^ Strategies to enhance fibrolysis are still lacking. Inhibiting lysyl oxidase‐like 2 (LOXL‐2) may aid in degradation of collagen fibres.^64^ With the recent understanding and developments in macrophage subsets to aid in fibrosis resolution, altering macrophage polarization and differentiation may potentially help in development of therapies targeting fibrolysis.^52^, ^65^

## 3 NASH DRUG DEVELOPMENT

Drug development not only involves identifying appropriate targets that will improve specific clinical endpoints, but must also ensure drugs are safe and efficacious with no harmful off target effects. The fundamental principle of drug approval is proving the safety and clinical benefit for patients with a specific condition. Drug development efforts have focused on showing clinical benefits either via hard outcomes or surrogate endpoints. A typical example of a hard outcome is achieving improvement in mortality due to a certain drug; however, this is quite challenging to do in chronic conditions such as NASH, as conducting studies require a long‐term and large numbers of patients. Therefore, such an approach carries a significant burden on sponsors and delays treatment discovery, which is an undesirable scenario in conditions with unmet needs such as NASH. Thus, the US Food and Drug Administration (FDA) created an alternative pathway known as the accelerated approval pathway (subpart H for drugs), which enables sponsors to apply for approval with trials of shorter duration using surrogate endpoints that may reflect clinical benefits.^66^ Almost all of the NASH drugs are currently under this pathway. Approval usually requires sponsors to study the drug further to prove its clinical benefits, especially when there is some uncertainty about the relationship between the surrogate endpoint and clinical benefits. Such studies are usually done in the postmarketing period.

### 3.1 Phase‐III clinical trials

#### 3.1.1 Obeticholic acid

Obeticholic acid (OCA) is an FXR agonist that has been tested in animal models as well as in humans with NASH.^19^ In the FLINT phase II study, patients with NASH and without cirrhosis were randomized to treatment with either OCA (25 mg) or placebo for 72 weeks. Patients randomized to OCA (45%) had improvements in liver histology (defined as a decrease in NAFLD activity score by at least two points without worsening of fibrosis) compared with patients in the placebo group (21%; relative risk 2.2, 95% CI 1.4‐3.3; *P* = .0002). Importantly, 35% of patients on OCA had improvements in fibrosis compared to 19% in the placebo group.^19^ Pruritus and changes in LDL were the main adverse events. The increase in LDL appears to be a class effect of FXR agonism.^67^ After this interim analysis, OCA was advanced to a phase III study (REGENRATE; NCT02548351). The primary efficacy analysis of REGENRATE included 931 NASH patients with stage 2 or 3 liver fibrosis and biopsies were analyzed after 18 months of treatments which included three groups, OCA 25 mg, OCA 10 mg or placebo OCA 25 mg met the preplanned primary endpoint of fibrosis improvement (≥1 stage) with no worsening of NASH using a planned 18‐month interim analysis (*P* = .0002).^68^ The FDA is currently discussing the approval of OCA.

#### 3.1.2 Cenicriviroc

Cenicriviroc (CVC) is a CCR2/CCR5 inhibitor that inhibits macrophage accumulation and improves fibrosis in animal models of NASH.^69^ In a phase‐IIb randomized, placebo‐controlled trial (CENTAUR), patients with NASH were randomized to CVC 150 mg QD or placebo.^50^ At year 1, 20% of NASH patients on CVC had ≥1 stage fibrosis improvement compared to 10% in placebo (*P* = .02). At year 2, there was no significant difference between CVC and placebo in achieving ≥1 stage improvement in fibrosis. Nevertheless, twice as many CVC patients who achieved ≥1 stage fibrosis improvement at year 1 maintained this benefit through year 2. The safety and tolerability of CVC were comparable to placebo over the 2‐year study. Currently, a phase‐III study (AURORA) is being conducted (NCT03028740) and CVC is also being tested in combination with tropifexor in a phase‐II study (see below).

#### 3.1.3 Elafibranor

Elafibranor is a peroxisome proliferator–activated receptor alpha and delta (PPAR‐α/δ) regulate lipid metabolism in liver and glucose homeostasis.^70^ The GOLDEN‐505 study was a phase‐IIb, randomized, placebo‐controlled trial that included 274 patients with NASH and no cirrhosis who were randomized to either elafibranor 80 or 120 mg/d, or placebo, for 52 weeks.^22^ The primary endpoint, reversal of NASH without worsening of fibrosis was not met though the definitions in this trial were slightly different from comparable studies. Therefore, a post hoc analysis of patients with baseline NAS ≥4 was performed and revealed a significant direct effect of elafibranor 120 mg vs placebo for the protocol‐defined and modified definitions. Gastrointestinal complaints and renal impairment were the main adverse events. RESOLVE‐IT is an ongoing phase‐III trial to evaluate the efficacy and safety of elafibranor in patients with NASH (NCT02704403).

#### 3.1.4 Belapectin

Belapectin (GR‐MD‐02) is a galectin‐3 inhibitor shown in NASH animal models to improve the disease activity and reduce or eliminate fibrosis.^71^ A recent phase‐IIb multicenter, randomized, double‐blind, placebo‐controlled study tested GR‐MD‐02 in 162 patients with NASH cirrhosis and portal hypertension (NCT02462967). A reduction in hepatic‐portal vein pressure gradient (HVPG) was the primary outcome, which was not met after 52 weeks of treatment. Hepatocyte ballooning was significantly reduced in the 2 mg/kg dose (*P* = .03) and only a trend was seen in the 8 mg/kg dose (*P* = .089). Although there were no significant benefits reported for both GR‐MD‐02 doses, some initial findings for patients with NASH and cirrhosis, without varices at baseline were of use.^72^ For this subgroup, GR‐MD‐02 2 mg/kg was associated with a significant effect on HVPG and lower incidence of varices development, but no change in liver fibrosis was seen. The investigators will launch a phase‐III study to look at this effect of GR‐MD‐02 in patients with NASH cirrhosis and portal hypertension without varices.^73^

#### 3.1.5 Resmetirom

Resmetirom (MGL‐3196), a thyroid hormone receptor‐β (THR‐β) agonist, was evaluated in a placebo‐controlled, multicenter, phase‐II trial in patients with liver‐biopsy‐confirmed NASH (NCT02912260). Patients received placebo or 80 mg MGL‐3196 once daily for 36 weeks, at week 4 ± 20 mg dose adjustment was added based on pharmacokinetics. The primary endpoint was met with 78 MGL‐3196‐treated patients showing a −36.3% relative and −7.6% absolute change from baseline measuring magnetic resonance imaging‐proton density fat fraction (MRI‐PDFF) at 12 weeks. A two‐point NAFLD activity score reduction with at least a one‐point reduction in ballooning or inflammation compared to placebo was seen (50.7% vs 32.4%; *P* = .09).^74^ MGL‐3196‐treated patients also saw a reduction in LDL‐C (*P* ˂ .0001), TG (*P* ˂ .0001), ApoB (*P* ˂ .0001) and ALT (40% reduction; *P* = .002) levels.^75^ Resmetirom (MGL‐3196) is now being investigated in a phase‐III clinical trial (NCT03900429).

#### 3.1.6 Aramchol

Aramchol inhibits stearoyl‐coenzyme A desaturase 1 (SCD1) which is one of the enzymes responsible for de novo lipogenesis (DNL) in the liver. In a phase‐IIA randomized, double‐blind, placebo‐controlled trial of 60 patients with biopsy‐confirmed NAFLD (six with NASH) for 12 weeks. MR spectroscopy measured liver fat content decreased by 12.57% in patients given 300 mg/d of aramchol, but increased by 6.39% in the placebo group (*P* = .02). Liver fat content decreased in the 100 mg aramchol group by 2.89%, but this change was nonsignificant (*P* = .35).^76^ This study led to the phase‐IIB (ARREST) study where patients were randomized to receive either 400 or 600 mg of aramchol or placebo per day. A significant reduction in liver fat was observed by MR spectroscopy in the 400 mg group (*P* = .045) and a trend seen in the 600 mg group (*P* < .066) compared with placebo which was the primary outcome. The investigators revealed an absolute reduction from baseline of 5% or higher in 47% of patients in the 600 mg group vs 37% in the 400 mg and 24% in the placebo.^77^ NASH resolution without worsening of fibrosis occurred more often in the 600 mg group than the placebo group (16.7% vs 5%, OR = 4.74; *P* = .0514. Currently, a phase‐III trial is being planned and will likely start by the time of publishing this review.

### 3.2 Phase‐IIb and phase‐IIa clinical trials

A plethora of novel investigational compounds for the treatment of NASH are currently being tested at phase‐IIa and phase‐IIb clinical trials.

#### 3.2.1 Farnesoid X receptor agonists

Cilofexor (GS‐9674) is a selective, nonsteroidal FXR receptor agonist and has shown to improve markers of cholestasis and liver injury.^78^ Patel et al^79^ presented data of a phase‐II randomized, controlled NASH clinical trial testing GS‐9674 (NCT02854605). The study included 140 NASH patients without cirrhosis treated with either 100 mg GS‐9674, 30 mg GS‐9674 or placebo, once daily for 24 weeks. MRI‐PDFF showed a 30% decrease in hepatic fat in 38.9% of patients on the 100 mg dose at week 24 compared to baseline, with 14% in the 30 mg treatment arm and 12.5% in placebo. Only 14% of patients in the 100 mg arm reported moderate to severe pruritus compared to 4% in the 30 mg and placebo groups. GS‐9674 did not alter serum lipids. GS‐9674 is now included in the combination therapy in the Gilead ATLAS study discussed below.

Tropifexor (TXR, also known as LJN452) is an FXR agonist currently in a two‐part phase‐IIb study to examine the safety, tolerability and efficacy of TXR (FLIGHT‐FXR, NCT02855164). The initial screening process testing the dose safety examined 77 patients with MRI‐PDFF ≥10 and histologic evidence of NASH or phenotypic diagnosis of NASH under randomization of four doses (10, 30, 60 and 90 μg of TXR with placebo).^80^ Part B isolated the 60 and 90 μg doses and tested a 12‐week treatment. Data indicate a reduction in hepatic fat with both doses and reduced ALT levels. Comparable rates of AEs including pruritus were reported for both TXR doses and placebo, with a mild dose‐response increase of LDL and decrease of HDL. The study has now advanced to its next stage with 150 patients diagnosed with histologic evidence of NASH (with F2 or F3) within 6 months of randomization.^80^

EDP‐305 is an FXR agonist that received a fast track status granted by the FDA for a phase‐IIa randomized, double‐blind, clinical trial (ARGON, NCT03421431). The safety, tolerability and pharmacokinetics of EDP‐305 will test two doses in 125 NASH patients for 12 weeks, and the results are expected to be presented in the near future.

#### 3.2.2 Fibroblast growth factor variants

BMS‐986036, also known as pegbelfermin, is a pegylated FGF21 analogue.^81^ A recent phase‐IIa study (NCT02413372) tested pegbelfermin in patients with NASH.^82^ In a 16‐week trial, a significant decrease was shown in absolute hepatic fat fraction in the group receiving 10 mg pegbelfermin daily (−6.8% vs −1.3%, *P* = .0004) and in the group receiving 20 mg pegbelfermin weekly (−5.2% vs −1.3%; *P* = .008) compared with the placebo. Pegbelfermin was generally well‐tolerated with only mild adverse events and no discontinuations or deaths. Further phase IIb is underway to test the safety and efficacy of pegbelfermin in adults with NASH and stage 3 and 4 liver fibrosis (FALCON1&2 studies: NCT03486899).

NGM282 is proving to be a safe and highly effective engineered FGF19 analogue in rapidly improving liver fat content and liver fibrosis in patients with NASH (NCT02443116).^18^ A recent randomized controlled study evaluated NASH patients who received NGM282 (1 mg, n = 24 or 3 mg n = 19) for 12 weeks.^83^ At week 12, 50% and 68% of the patients receiving NGM282 1 or 3 mg, respectively, improved histological NAFLD activity score by two or more points without worsening fibrosis. Compared to baseline, liver fibrosis improved by one stage or more in 25% and 42% with 1 or 3 mg NGM282, respectively. A reduction in absolute MR liver fat content was seen as early as week 6, and by week 12, 92% and 100% of patients receiving 1 or 3 mg NGM282, respectively, achieved a clinically meaningful ≥5% reduction in absolute liver fat content. In some patients, serum levels of LDL‐C increased at week 2.^83^ These encouraging results were accompanied by very few adverse events, which were either mild or moderate in severity. These promising results should enable further phase IIb and even phase III studies to develop for NGM282.

#### 3.2.3 Glucagon‐like peptide‐1 targeted drugs

Glucagon‐like peptide‐1 (GLP‐1) receptor agonists, also known as incretin mimetics, are agonists of the GLP‐1 receptor. FDA‐approved drugs already exist for the treatment of type 2 diabetes, with significant effects on weight loss and cardiovascular outcomes, but their safety and efficacy are now being tested in clinical trials for NASH.^84^, ^85^ The proposed mechanism on NASH is related to weight loss, yet others are being explored. Liraglutide is a GLP‐1 receptor agonist already approved for the treatment of diabetes and has undergone phase‐II trials showing improved liver histology in patients with NASH (LEAN, NCT01237119). The primary outcome of NASH resolution was achieved in 39% of patients who received liraglutide compared to 9% in the placebo group in a 48‐week study. Adverse events were gastrointestinal disorders.^11^ Similar findings were confirmed in the Lira‐NAFLD study looking at liver fat content in type 2 diabetes patients. A marked relative reduction in liver fat content of 31% (*P* ˂ .0001) was seen (NCT02048189).^86^

Semaglutide is another GLP‐1 analogue in ongoing phase‐II studies to explore the safety, tolerability and efficacy of semaglutide as a single‐use drug in NASH.^87^ A potential combined therapy with firscocostat and cilofexor (discussed below) is also in the pipeline.

#### 3.2.4 Fatty acid synthesis inhibitors: acetyl‐CoA carboxylase inhibitors

Acetyl‐CoA carboxylase (ACC) catalyses the rate‐limiting step in DNL; thus, ACC inhibition improves steatosis, inflammation and fibrosis in preclinical models.^88^ GS‐0976 (firsocostat) is a liver‐directed inhibitor of ACC, shown to reduce DNL and liver fat in a proof‐of‐concept study of NASH patients. A randomized, placebo‐controlled study tested 126 noncirrhotic patients with hepatic steatosis of at least 8% based on MDRI‐PDFF and liver stiffness of at least 2.5 kPA based on magnetic resonance elastography or liver biopsy (NCT02856555). The 20 mg dose of GS‐0976 proved more efficacious than the 5 mg and placebo, with 48% of patients having a relative decrease of at least 30% from baseline in MRI‐PDFF compared to placebo (*P* = .004) by 12 weeks. The 20 mg dose also had a greater median relative decrease in MRI‐PDFF compared to placebo (*P* = .002).^89^

Also, in the pipeline for ACC inhibitors is PF‐05221304, a potent, selective and reversible dual ACC 1/2 inhibitor designed to have asymmetric distribution in the liver. Initial safety, tolerability and pharmacokinetics of PF‐05221304 findings were presented recently. PF‐05221304 was well‐tolerated at all single and multiple oral doses.^90^ It has now been fast‐tracked granted by the FDA to phase‐IIa clinical trial (NCT03248882).

In reducing DNL via ACC inhibition, one of the side effects may be a potential worsening of the blood lipid profile. Both proof‐of‐concept studies as well as early‐stage clinical trials demonstrated an increase in the serum triglyceride levels in patients receiving ACC inhibitors.^89^, ^91^

#### 3.2.5 Peroxisome proliferator–activated receptors‐α/γ/δ agonists

Seladelpar (MBX‐8025), a selective PPAR‐δ agonist, has been shown to reverse NASH pathology in diabetic mouse models.^92^ Currently, a phase‐IIb randomized, placebo‐controlled study is testing three doses for 52 weeks in patients with liver‐biopsy NASH (NCT03551522). Saroglitazar is a dual PPAR‐α/γ agonist which has undergone various testing in rodent models and shows promise for human subjects with NASH and diabetes.^93^, ^94^ The EVIDENCES IV phase‐II clinical trial (NCT03061721) is currently being run as a randomized, placebo‐controlled study in 104 patients. Lanifibranor (IVA337) is a moderately potent and a well‐balanced pan PPAR‐α agonist.^95^ A phase‐IIb NATIVE study is underway (NCT03008070), with a second recruiting type 2 diabetes mellitus patients with NAFLD (NCT03459079).

#### 3.2.6 Mitochondrial pyruvate carrier

MSDC‐0602K is being tested in phase‐II trials. The EMMINENCE phase‐IIb trial (NCT02784444) is a 12‐month randomized study to evaluate the safety, tolerability and efficacy of three doses of MSDC‐0602K in patients with NASH but no cirrhosis. The primary outcome is improved NAFLD activity score defined as a decrease of at least two points with no worsening of fibrosis stage at 12 months. Initial reports confirm significant improvements in liver enzymes and markers of fibrosis after 6 months of treatment.^96^

#### 3.2.7 Thyroid hormone receptor

VK2809‐201 is a novel liver‐directed THR‐ß agonist. Currently, in a phase‐IIa randomized, placebo‐controlled trial of patients with NAFLD with LDL‐C >110 mg/dL and liver fat content >8% (PDFF). Preliminary data show significant reductions from baseline in LDL‐C. Mean absolute percentage change in liver fat at 12 weeks was significant in both treatment arms (10 mg QOD, *P* = .011; 10 mg QD, *P* = .0025) with a median relative percentage change of −56.5% (10 mg QOD) and −59.7% (10 mg QD) compared to placebo (−8.9%).^97^

### 3.3 Drugs that have failed clinical trials

Despite targeting the mechanistic pathways, a number of drugs have failed to demonstrate clinical benefit in targeting the pathways mentioned above. Simtuzumab, a monoclonal antibody against LOXL‐2, failed to demonstrate benefit in patients with either bridging fibrosis or compensated cirrhosis.^98^ Selonsertib, an ASK1 inhibitor, was not superior to placebo in improving fibrosis in patients with both bridging fibrosis and cirrhosis.^99^ Emricasan, a pan‐caspase inhibitor, did not meet the primary endpoints of improvement in portal hypertension or fibrosis in pre‐cirrhotic NASH patients.^100^ Despite the failures, some of these drugs are being tested in subgroups of NASH patients, or in combination regimens with other agents (see below).

### 3.4 Combined therapies for NASH

Due to the complexity of NASH pathogenesis, there is no ‘one drug fits all’ approach. These pathophysiological pathways may vary among patients, generating subtypes of NASH. Understanding a patient's subtype could lead the way to personalized medicine approaches.^101^, ^102^ Until then, several trials are trying a combined therapy approach to target several pathways at one time to generate an effective treatment for NASH.

A proof‐of‐concept combined therapy study using the nonsteroidal FXR agonist cilofexor (GS‐9674) and the ACC inhibitor firsocostat (GS‐0976) was launched in patients with NASH. MRI‐PDFF measured a significant decline of at least 30% in hepatic fat from baseline to 12 weeks observed in 74% of patients.^103^ In addition, Gilead is testing this combination in a triple form with the inclusion of selonsertib (GS‐4997), an apoptosis signal‐regulating kinase 1 (ASK1) inhibitor in the ATLAS trial (NCT03449446). Similarly, cilofexor (GS‐9674) and firsocostat (GS‐0976) are being tested with semuglutide in a proof‐of‐concept, open‐label study evaluating the safety, tolerability and efficacy of monotherapy and combination regimens in subjects with NASH (NCT03987074).

The TANDEM phase‐IIa clinical trial study is combining tropifexor (LJN452) and CVC, to test the safety, tolerability and efficacy in NASH patients with liver fibrosis (NCT03517540).

Lastly, a phase IIa in NAFLD patients will test the pharmacodynamics, safety and tolerability in a double‐therapy of PF‐05221304 and PF‐06865571 for 6 weeks (NCT03776175).

**Figure 2:**
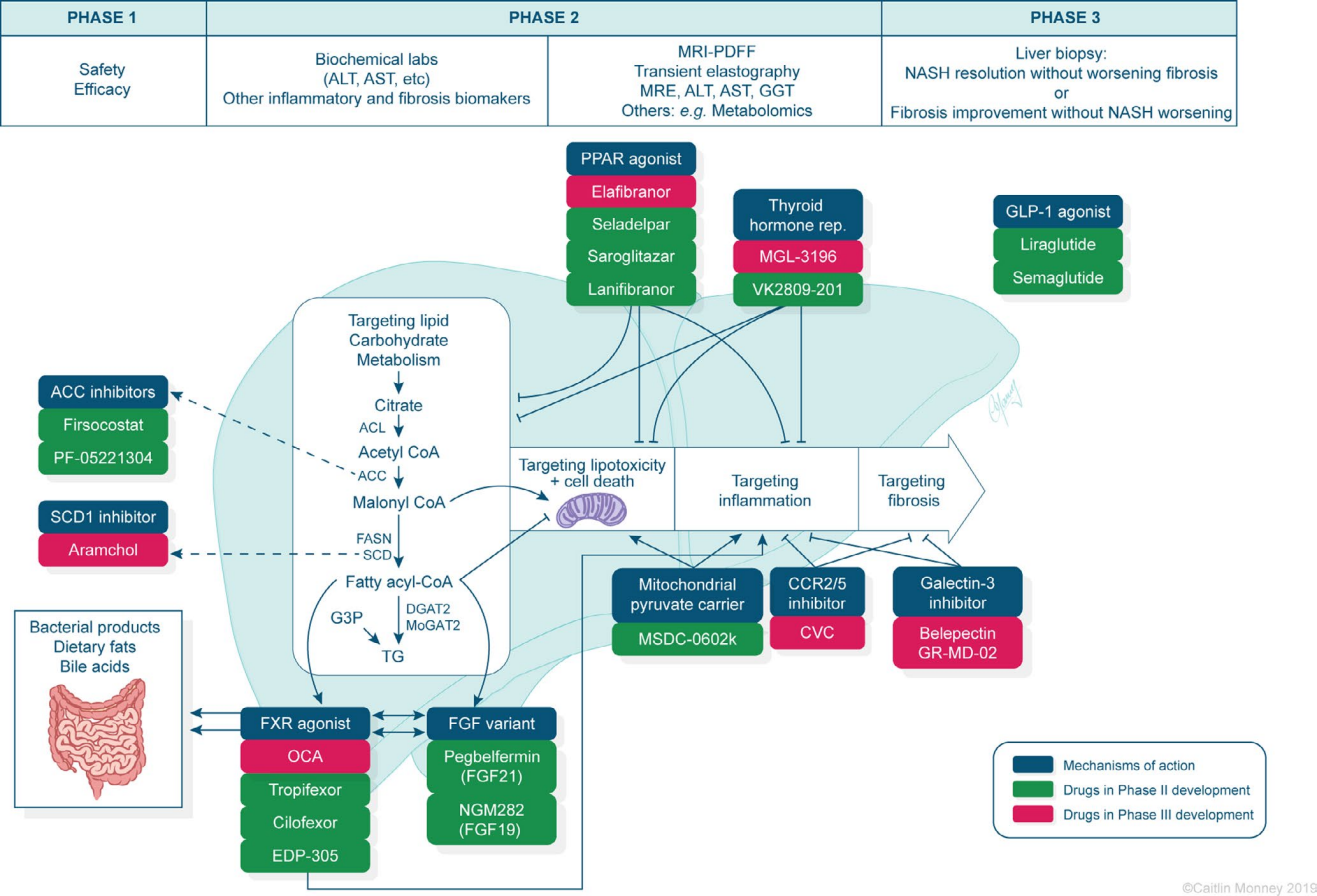
Mechanism of action of drugs currently in phase‐II and phase‐III development. ACC, acetyl Co‐A carboxylase; ACL, adenosine triphosphate‐citrate lyase; ALT, alanine aminotransferase; AST, aspartate aminotransferase; CCR, C‐C motif chemokine receptor; CVC, cenicriviroc; DGAT2, diacylglycerol O‐acyltransferase 2; FASN, fatty acid synthase; FGF, fibroblast growth factor; FXR, farnesoid X receptor; G3P, glycerol‐3‐phosphate; GGT, gamma‐glutamyl transpeptidase; GLP‐1, glucagon‐like peptide‐1; MoGAT2, monoacylglycerol O‐acyltransferase 2; MRE, magnetic resonance elastography; MRI‐PDFF, magnetic resonance imaging proton density fat fraction; NASH, nonalcoholic steatohepatitis; OCA, obeticholic acid; PPAR, peroxisome proliferator‐activated receptor; SCD1, stearoyl‐CoA desaturase 1; TG, triglyceride; thyroid hormone rep., thyroid hormone receptor

These drugs and trials have been summarized in Table 1 and Figure 2.

**Table 1:** List of drugs currently being evaluated in clinical trials

Drug(s)	Mechanism of action	Phase in clinical trial	Trial identification
Obeticholic acid (OCA)	Farnesoid X receptor (FXR) agonist	III	NCT02548351
Cenicriviroc (CVC)	CCR2/CCR5 inhibitor	III	NCT03028740
Elafibranor	PPAR‐α/δ agonist	III	NCT02704403
Belapectin (GR‐MD‐02)	Galectin‐3 inhibitor	III	4th quarter 2019
Resmetirom (MGL‐3196)	Thyroid hormone receptor ß (THR‐ ß) agonist	III	NCT03900429
Aramchol	Stearoyl‐CoA desaturase 1 (SCD1) inhibitor	III	3rd quarter 2019
Cilofexor (GS‐9674)	FXR agonist	II	NCT02854605
Tropifexor (TXR/LJN452)	FXR agonist	IIb	NCT02855164
EDP‐305	FXR agonist	IIa	NCT03421431
Pegbelfermin (BMS‐986036)	Fibroblast growth factor 21 (FGF21) analogue	IIb	NCT03486899
NGM282	FGF19 analogue	IIb	NCT03912532
Liraglutide	Glucagon‐like peptide‐1 (GLP‐1) agonist	II	NCT01237119
Semaglutide	GLP‐1 agonist	II	NCT03987451
Firsocostat (GS‐0976)	Acetyl‐CoA carboxylase (ACC) inhibitor	II	NCT02856555
PF‐05221304	ACC inhibitor	IIa	NCT03248882
Seladelpar (MBX‐8025)	PPAR‐δ agonist	IIb	NCT03551522
Saroglitazar	PPAR‐α/γ agonist	II	NCT03061721
Lanifibranor (IVA337)	PPAR‐α agonist	IIb	NCT03008070
MSDC‐0602K	Mitochondrial pyruvate carrier (MPC) inhibitor	IIb	NCT02784444
VK2809‐201	THR‐ß agonist	IIa	NCT02927184
Cilofexor (GS‐9674) + Firsocostat (GS‐0976)	FXR agonist + ACC inhibitor	II	NCT02781584
Cilofexor (GS‐9674) + Firsocostat (GS‐0976) + Selonsertib	FXR agonist + ACC inhibitor + apoptosis signal‐regulating kinase 1 (ASK‐1) inhibitor	II	NCT03449446
Cilofexor (GS‐9674) + Firsocostat (GS‐0976) + Semaglutide	FXR agonist + ACC inhibitor + GLP‐1 agonist	II	NCT03987074
Tropifexor (LJN452) + and Cenicriviroc (CVC)	FXR agonist + CCR2/CCR5 inhibitor	II	NCT03517540
PF‐05221304 + PF‐06865571	ACC inhibitor + Diacylglycerol O‐Acyltransferase 2 (DGAT2)	II	NCT03776175

## 4 PRACTICAL CONSIDERATIONS IN THE USE OF DRUG THERAPY FOR NASH IN THE CLINIC

While many drugs are in the development pipeline, there are currently no drugs approved for the treatment of NASH. This creates a serious challenge for the clinician faced with a patient with NASH who has fibrosis stage 2 or higher which is associated with increased all‐cause and liver‐related mortality.^104^ We describe below our current approach to such patients. It must be emphasized that these do not represent a consensus statement and the use of specific drugs must be considered ‘off‐label’ use despite the evidence for benefit that we will use to support our practice.

### 4.1 Goals of treatment

An important aspect of NASH management is that it is part of a multisystem disease which affects the heart, arteries, liver, kidneys, striated muscle and the pancreas.^105^ Afflicted patients usually have one of more of these end‐organs affected, there is a collinearity between the stage of liver disease and other end‐organ disease.^106^, ^107^ This leads to multiple competing risks from different end‐organ involvement such as coronary artery disease, heart failure with preserved ejection fraction, chronic kidney disease and liver disease specifically the development of cirrhosis. Given that these risks are present simultaneously, treating one without the others is not likely to improve patient outcomes. It is therefore critically important to assess the status of all of the end‐organs involved in all patients with NAFLD especially NASH and development a comprehensive management strategy that protects the patient from all of these competing threats to life while minimizing the burden of testing and drugs that the patient is exposed to. NASH is also associated with an increased risk of mortality from cancers including extrahepatic cancers.^108^ It is also therefore relevant to follow current guidelines for cancer screening as appropriate.

### 4.2 Treating the root cause

There are two philosophical approaches to the treatment of NASH. The first is the treatment of the root cause which is excess adiposity and systemic inflammation which delivers a pro‐inflammatory soup of fatty acids, sugars and inflammatory cytokines to the liver. Alternately, an approach including specific anti‐inflammatory or antifibrotic therapies to slow down the progression to cirrhosis or even reverse cirrhosis are being attempted. Given the multiplicity of down‐stream pathways involved in inflammation and fibrosis, the latter strategy has yet to demonstrate significant benefit. On the other hand, medically induced weight loss and bariatric surgery have both shown benefit.^109^, ^110^ Weight loss also improves cardio‐metabolic outcomes risk.^111^, ^112^ It is therefore the corner‐stone of the management of patients with NAFLD including NASH.

A key question is as follows: How much weight loss is needed to produce clinically meaningful benefit? It is widely touted that 5% weight loss improves steatosis, 7% weight loss resolves NASH and 10% weight loss or more causes fibrosis to regress.^113^ A critical review of the literature reveals this to be a false statement. In a major study that is cited to support these thresholds, many individuals with 7% weight loss did not experience resolution of steatohepatitis.^114^ Thus, this threshold is not specific. On the other hand, many patients with <10% weight loss experienced fibrosis improvement. These data indicate that there are factors other than weight loss that are also relevant in the liver outcomes of weight loss. Importantly, it must be noted that long‐term reduced progression to cirrhosis or cirrhosis reversal have not been demonstrated with medical weight loss. Further, it is unclear whether a 10% weight loss in a 350 lb individual has the same biological impact as a 10% weight loss in a 250 lb individual. Despite these uncertainties, there is a large body of literature documenting the benefits of weight loss in general. An attempt should therefore be made to reduce adiposity and bring it towards a more physiological state.

It is also well known that only a minority of individuals can lose weight and sustain the weight loss. The reasons for this are multifactorial, and unfortunately, many healthcare providers do not have the expertise or time to unravel these and address the relevant factors that are barriers to weight loss. It is our experience that a clinical psychologist can play a major role in helping dissect the reasons for weight gain including the presence of eating disorders, eating to resolve stress, food addictions, impulsivity, work and social and economic factors. In our view, this is a critical part of managing such patients to maximize sustained weight loss.

Another key factor limiting the success of medical weight loss approaches is the expectation that the patient will change their food habits, while the rest of the family does not. We have recently found that in those with NASH cirrhosis, a large number of caregivers also have the condition and this is almost certainly linked to common lifestyle‐related risk factors.^115^ It is therefore important to engage the entire family unit in changing lifestyle.

While there is debate about whether isometric or isotonic exercise is good, from a practical point of view, both are better than not exercising. We recommend that patients start an exercise programme initially under supervision especially if they have long‐standing diabetes, hypertension, CKD or peripheral vascular disease. The level of exercise should be slowly ramped up with positive feedback and support provided to the patient.

There is also substantial debate over diet. While the Mediterranean diet is widely touted, we find that the acceptance of such diet varies with the cultural and ethnic background of our patients.^116^, ^117^ It is our experience that dietary guidance is best received when provided in the patients social, cultural and economic context in language that is translatable in to action by the patient. Given the time constraints on many physicians, specific counselling by a nutritionist is often valuable in this regard.

In those who are unable to lose weight with medical advice, drug therapy may be considered. There are several drugs that are currently available for weight loss. It is our approach to use drugs that not only lead to weight loss but also are known to improve cardio‐metabolic outcomes and potentially benefit NASH as well. We therefore tend to use a GLP‐1 agonist such as liraglutide which has been shown to improve all‐cause mortality in a high‐risk population of patients with type 2 diabetes and also improve liver histology in a small phase‐II proof‐of‐concept trial.^11^ Semaglutide is another approved agent that has been shown to produce substantial weight loss, improve insulin sensitivity and reduce cardiovascular and all‐cause mortality in a relevant population.^118^, ^119^ Recently, a hydrogel which expands in the stomach and induces satiety has been approved by the FDA (Gelesis100). This appears to an extremely safe and well‐tolerated treatment of obesity and is not associated with nausea at levels seen with GLP‐1 agonists.^120^, ^121^ Bariatric surgery is considered in those with other comorbidities that warrant consideration for such surgery. It is important to assess hepatic risk and overall risk of surgery before recommending these procedures.

### 4.3 Control of diabetes

It is recommended that diabetes be adequately controlled and the proportion of patients with a HbA1c below 8.5% (69 mmol/mol) is considered a quality metric linked to physician reimbursement. This is often achieved by the use of insulin which is started after first line treatment with metformin. For the patient with NASH, the associated weight gain with insulin creates a management conundrum; this is especially true for patients with cirrhosis who are given tight glycaemic control and weight loss targets to qualify for listing for transplant at many centres. It is important to note that obese NASH cirrhotic patients also have sarcopenia and are most insulin resistant and metabolically inflexible.^122^, ^123^ They often require more aggressive hypoglycaemic therapy to achieve glycaemic targets but this can worsen adiposity. The use of GLP‐1 and SGLT‐2 inhibitors which have both been shown to cause weight loss, improve survival and potentially stabilize eGFR is preferable over insulin in our view, and we reserve insulin as back‐up therapy after initiation of these agents in those with NASH cirrhosis.

### 4.4 Atherogenic dyslipidaemia

Given the increased risk of cardio‐metabolic outcomes in this population, it is important to assess the lipid profile in patients with NAFLD. The potential risks of hepatotoxicity from statins are overestimated, and statins are by and large safe to use in this population.^124^ In the absence of documented statin toxicity, statins should not be withheld if indicated. In patients with NASH, excess triglyceride from the liver comes out in VLDL forming large VLDL particles. These are incompletely hydrolyzed by lipoprotein lipase in the periphery resulting in intermediate density lipoproteins and LDL particles with excess triglycerides which are susceptible to hepatic lipases. These cleave LDL particles resulting in a large number of small dense LDL particles which are more atherogenic than large LDL.^125^ Together with increased hepatic cholesterol synthesis and decreased hepatic LDL receptor expression, these link NASH to increased risk of atherogenesis.^126^ Statins reduce LDL cholesterol but do not impact small dense LDL cholesterol particle number or concentration.^127^ We recommend the use of fibrates with a statin when there is a suboptimal decrease in LDL‐C or high levels of small dense LDL or when there is accompanying hypertriglyceridaemia. PCSK9 inhibitors may be considered in those with a high coronary event risk and who are intolerant of statins or do not achieve adequate response to maximal statin doses.^128^

### 4.5 Treatment of the NASH itself

In many instances, the treatment of the root cause is associated with de‐fatting of the liver, normalization of liver enzymes and a decrease in liver stiffness measured by fibroscan. Given the variability of fibroscan readings over time, it is our approach to use 2D MRE to follow those who have been initiated on drug treatment. In selected individuals, with stage‐3 fibrosis or higher, we use vitamin E (400‐800 IU/d of rrr alpha tocopherol). This has been shown to reduce disease activity and in those with a two point or greater decrease in the NAFLD activity score, fibrosis also tends to improve.^35^, ^129^ The potential risks of vitamin E use have been largely debunked, and its use must also take in to consideration the potential risk of leaving NASH untreated vs the risks of the drug. Further, vitamin E primarily benefits those with a haptoglobin 2 genotype, and we use this to also determine who to treat.^130^ This is not however routinely available in clinics, and this represents something that is currently available at our centre on a research‐basis only.

There are also several studies demonstrating the benefit of pioglitazone a PPAR‐γ agonist. It improves steatohepatitis, disease activity scores and also may improve fibrosis.^10^ Its use is limited by weight gain and is therefore often used as a second‐line treatment.

### 4.6 Summary

The optimal pharmacological treatment of NASH must be considered in the context of the disease stage and the comorbidity profile and status of key extrahepatic end‐organs. The treatment of the root cause of the disease, that is, excess adiposity without losing muscle mass is a central theme for the management of these patients. Several recently approved agents demonstrate not only weight loss but improve all‐cause mortality and cardiovascular outcomes in high‐risk overweight/obese individuals and can be considered if the patient has excess adiposity. Specific anti‐NASH agents can be used to complement these approaches. As new drugs are approved for NASH, their use will need to be integrated in to the overall care of this multisystem disorder.

## CONFLICT OF INTEREST

Arun Sanyal: Dr Sanyal is President of Sanyal Biotechnology and has stock options in Genfit, Akarna, Tiziana, Indalo, Durect and Galmed. He has served as a consultant to Astra Zeneca, Nitto Denko, Enyo, Ardelyx, Conatus, Nimbus, Amarin, Salix, Tobira, Takeda, Jannsen, Gilead, Terns, Birdrock, Merck, Valeant, Boehringer‐Ingelheim, Lilly, Hemoshear, Zafgen, Novartis, Novo Nordisk, Pfizer, Exhalenz and Genfit. He has been an unpaid consultant to Intercept, Echosens, Immuron, Galectin, Fractyl, Syntlogic, Affimune, Chemomab, Zydus, Nordic Bioscience, Albireo, Prosciento, Surrozen and Bristol Myers Squibb. His institution has received grant support from Gilead, Salix, Tobira, Bristol Myers, Shire, Intercept, Merck, Astra Zeneca, Malinckrodt, Cumberland and Novartis. He receives royalties from Elsevier and UptoDate. Mazen Noureddin: Dr Noureddin has been on the advisory board for Gilead, Intercept, Pfizer, Novartis, Blade, EchoSens North America, OWL, and Abbott. He has received research support from Allergan, BMS, Gilead, Galmed, Galectin, Genfit, Conatus, Enanta, Novartis, Shire and Zydus. He is a minor shareholder or has stocks in Anaetos and Viking. Mark Muthiah: No conflict of interests to declare.

## AUTHORS' CONTRIBUTIONS

Mazen Noureddin and Mark D. Muthiah involved in writing of original draft, review and editing and visualization and Arun J. Sanyal involved in conceptualization, supervision and writing of original draft, review and editing.

## ETHICAL APPROVAL

The authors declare that the manuscript is original and has not been submitted for publication elsewhere. No specific ethical approval or informed consent was required for this review article.

## Data Availability

Data sharing is not applicable to this article as no new data were created or analysed in this study.
